# Clinical predictors and antibiotics sensitivity of bacterial pathogens of pharyngotonsillitis among children aged 1-17 years in a Tertiary Hospital, Abakaliki, Southeast, Nigeria

**DOI:** 10.11604/pamj.2025.51.51.36632

**Published:** 2025-06-18

**Authors:** Sunday Ogo Nweke, Maria-Lauretta Orji, Chijioke Ogodo Ogeh, Theresa Nwamaka Nnaji, Thecla Chinonyelum Ezeonu, Michael Akinwale Efunshile

**Affiliations:** 1 Department of Paediatrics, Alex Ekwueme Federal University Teaching Hospital Abakaliki, Ebonyi State, Nigeria,; 2 Ebonyi State University, Abakaliki, Nigeria

**Keywords:** Antibiotics sensitivity, bacterial pathogens, clinical predictors, pharyngotonsillitis

## Abstract

**Introduction:**

clinical presentations that possibly predict bacterial pharyngotonsillitis are invaluable in early institution of care, so as to reduce morbidity from the disease. This is augmented by knowledge of antibiotic sensitivity pattern of the commonly isolated pathogens in the locality. The study was aimed at determining the relationship between clinical presentations and laboratory diagnosis of bacterial pharyngotonsillitis and to determine the antibiotics susceptibility pattern of the isolates.

**Methods:**

a hospital-based descriptive cross-sectional study was carried out over period of seven (7) months from 3^rd^ November, 2020, to 28^th^ May, 2021. The participants were consecutively recruited. Structured questionnaire was used to obtain information on socio-demographics of the participants. Throat swabs were taken from participants and sent to Medical Microbiology Research Laboratory of Alex Ekwueme Federal University Teaching Hospital, Abakaliki for microscopy, culture and sensitivity. Descriptive statistical analysis was used to determine frequencies and percentages. Univariate analysis was used to find any association between dependent variable and independent variables.

**Results:**

of the 150 participants that were recruited, 58.7% were males, giving a male-to-female ratio of 1.4: 1. The median age of participants was 5.0 years with an interquartile range of 3-8 years. Bacterial isolates were noted in 54 (36%). There was an overlap between the clinical features seen in participants with positive bacteria culture and those seen in negative (sterile) culture. The positive predictive values using clinical features to predict pharyngotonsillitis of bacterial origin were low. Most of the isolated organisms were more susceptible to cephalosporins (ceftriaxone and cefuroxime) than the penicillin (penicillin V and amoxicillin clavulanate).

**Conclusion:**

an overlap observed between the clinical presentations of the participants with positive throat culture and those without in this study makes clinical features unreliable in predicting pharyngotonsillitis of bacterial origin. Hence routine throat swab for bacteriology culture should be done for all children presenting with features suggestive of pharyngotonsillitis before commencement of antibiotics. However, where such services are not readily available, the use of cephalosporins empirically is recommended.

## Introduction

Pharyngotonsillitis is a common upper respiratory tract infection in children [1]. It is the second most common cause of childhood respiratory diseases with a high morbidity and mortality globally [2]. It is often characterized by fever, sore throat, enlarged and hyperaemic tonsils with or without exudates and tender cervical lymphadenopathy [3,4]. The clinical practice guideline by the Infectious Diseases Society of America states that clinical features such as rhinorrhea, cough, oral ulcers in the presence of fever and sore throat suggest viral aetiology [5]. However, Shaikh *et al*. [6] noted an overlap in the clinical presentations found in pharyngitis and tonsillitis of either viral or bacterial origin. There are contrasting views on the use of clinical signs and symptoms in predicting bacterial pharyngotonsillitis, while some literature agreed on its use others did not. Hence the need to assess these clinical predictors as they may be of help in timely management of bacterial pharyngotonsillitis. Recent reports of the antimicrobial stewardship programme in Nigeria showed that irrational use of antibiotics is common among Nigerian clinicians and these antibiotics are mostly prescribed without sensitivity test report and this has contributed significantly to bacterial antibiotic resistance [7,8].

Acute bacterial pharyngotonsillitis is a known risk factor for acute glomerulonephritis which is responsible for about 7.9% of renal diseases [9,10]. Furthermore, rheumatic fever, a known non-suppurative complication of bacterial pharyngotonsillitis has continued to constitute a significant health concern around the world, especially in developing nations [11,12]. It is estimated that there are over 15 million cases of rheumatic fever and rheumatic heart disease worldwide, with 282,000 new cases and 233,000 deaths annually and most of these morbidities and mortalities occur in the developing nations, Nigeria inclusive [13,14]. The variation in the pattern of these morbidities and mortalities are affected by different climatic, demographic and socio-economic factors [15]. Hence, the need for prompt diagnosis and appropriate treatment of bacterial pharyngotonsillitis cannot be over-emphasized. Early diagnosis and prompt treatment of bacterial pharyngotonsillitis will help to prevent rheumatic fever, rheumatic heart disease and kidney injury as well as peritonsillar and retropharyngeal abscesses. This study is therefore aimed at determining the clinical presentations that can predict bacterial pharyngotonsillitis and antibiogram of pharyngotonsillitis of the bacteria isolate. It is hoped that the findings from this study will contribute positively to the management of bacterial pharyngotonsillitis and subsequent reduction in post-bacterial pharyngotonsillitis-related complications and may provide basis for commencement of empirical antibiotic treatment for children with pharyngotonsillitis of bacterial origin.

## Methods

**Study design:** descriptive cross-sectional study was conducted to determine the clinical presentations that can predict bacterial pharyngotonsillitis and antibiogram of the bacteria isolates.

**Study setting and population:** the study was carried out in Abakaliki, the capital city of Ebonyi State, Nigeria. Nigeria is a country in the West African region. Ebonyi State is one of the 5 States in the South-Eastern part of Nigeria. It is located in the rain forest zone; the climate is tropical. The annual rainfall varies from 2,000mm in the Southern areas to 1,150mm in the north. The temperature throughout the year ranges between 21°C to 29°C. It has two seasons, dry and wet season. The dry season lasts from November to March while the rainy season lasts from April to October [16]. It was created in 1996 and has 13 Local Government Areas (LGA). Abakaliki is located at latitude 6.32°N and longitude 8.11°E at an elevation of 65 metres above sea level with a population of 134,102 making it the largest city in Ebonyi state [17]. The metropolis comprises two Local Government Areas (LGA), namely Abakaliki and Ebonyi. It has an area of 452sq kilometres. The inhabitants of the area are mainly of the Igbo ethnic group. They are mainly farmers and traders while some are civil servants.

The study populations were 150 children aged 1-17 years seen at children outpatient clinic (CHOP), in Alex Ekwueme Federal University Teaching Hospital Abakaliki, Ebonyi State over a period of seven (7) months from 3^rd^ November, 2020 to 28^th^ May, 2021. Every participant that met the study inclusion criteria which were children aged 1-17 years that presented at children outpatient clinic of Alex Ekwueme Federal University Teaching Hospital Abakaliki with a history of sore throat and or evidence of inflamed or with exudates on the pharynx/tonsils with any of the following symptoms and signs: difficulty / painful swallowing, fever (recorded temperature = 38°C), vomiting, cough and anterior cervical adenitis (tender and palpable anterior cervical lymph nodes) were consecutively recruited until the required sample size was met [18]. Those that met the inclusion criteria but had received antibiotics within 72 hours of presentation to the hospital and those with signs and symptoms suggestive of acute epiglottitis such as stridor, difficulty in breathing and tripod position were excluded.

**Variables:** variables included sore throat, vomiting, cough, fever (recorded temperature = 38°C), difficulty / painful swallowing, hypereamic tonsils/pharynx, exudative pharynx/tonsils, and anterior cervical lymph node enlargement.

### Data resources and measurement

**Data collection tool:** structured questionnaire [19], swab stick, pen touch, disposable hand gloves, face masks and wet ‘amies’ transport medium (Copan Tran system TM Brescia, Italy).

**Data collection:** demographic variables such as gender, age, education, parents' occupations and their educational qualifications were collected using the questionnaire. Socio-economic status was calculated using the method described by Olusanya *et al*. [20]. Other variables collected with the structured questionnaire were sore throat, exudates on the pharynx/tonsils, hyperaemic pharynx/tonsils, difficulty/painful swallowing, fever (recorded temperature = 38°C), vomiting, cough and anterior cervical lymph node enlargement.

**Specimen collection and laboratory procedure:** throat swab samples from pharynx and tonsils were collected by the researcher with the help of the two trained assistants. Hands were thoroughly washed, gloves and face masks were worn. Younger participants were made to sit comfortably in their mothers' laps while the adolescents sat on the consulting chair. Both were made to face a strong light source to ensure the area to be swabbed was visible. Package containing the swab stick was aseptically opened. The head of the patient was tilted backward and stabilized by a trained assistant, then patient was asked to open his/her mouth and stick out his/her tongue (for co-operative child) while wooden tongue depressor was used to open the mouth and hold the tongue in place (for an uncooperative child), a flashlight (pen torch) was used to light up the back of the throat by the second assistant. The inflamed, hyperaemic and purulent pharynx and tonsils were swabbed using the sterile cotton tipped applicator by the researcher. Necessary care was taken not to swab the cheeks, tongue, lips or other areas of the mouth. The swab was placed immediately in the wet ‘amies’ transport medium (Copan Tran system TM Brescia, Italy) and transported by the researcher within an hour to the microbiology research laboratory at the AE-FUTHA after proper labelling. On arrival at the laboratory the samples were inoculated into the sheep blood agar media within 5-10 minutes and incubated in the incubator. The isolated organisms were tested for antibiotic susceptibility.

**Antibiotic sensitivity test:** the Kirby-Bauer agar diffusion disk method was aseptically employed in the antibiotic sensitivity test [21]. Gram staining to confirm culture purity from the subculture plate was performed. The isolated suspension was then compared to get a turbidity equivalent of 0.5 McFarland standard [21]. The entire surface of Mueller Hinton Agar (MHA) was streaked 3 times with the wet swab, turning the plate 60 degrees between streaking to obtain an even inoculation. Antibiotics-impregnated disk were then applied on the inoculated plates within 15 minutes. The agar plates were then placed inverted and incubated at 37°C for 24hrs. After the 24hrs incubation, the plates were then removed from the incubator and zones of inhibition around the antibiotics were measured in millimeter (mm) using metric rule and the results were recorded. The measured inhibition zone (zone diameter break point) was recorded as either sensitive (S), intermediate (I) or resistant (R) after comparing with the Clinical and Laboratory Standards Institute (CLSI) chart. The antibiotics tested were: penicillin V, flucloxacillin, cefuroxime, ceftriaxone, ciprofloxacin, amoxicillin-clavulanate, azithromycin, bacitracin and optochin, which were selected based on the literature and local clinical experience.

**Sample size:** the sample size of 150 was arrived at using the formulae [22]:

n = z^2^pq/d^2^

where: n = sample size when the population is > 10,000 and n_f_= n/1+n/N [22]

where: nf = desired sample size when population was less than 10,000. Consecutive sampling technique was employed until the required sample size was achieved.

**Data analysis:** all data collected through the questionnaire were entered into Statistical Package for Social Science (SPSS) Window Version 25.0 (Armonk, NY: Corp, 2017). A double check was adopted on entry of the data to ensure higher degree of accuracy. The quantitative variables (age and temperature in degree Celsius) were categorized and its summary together with other categorical variables (sex, social status, and symptoms and signs such as sore-throat, cough, fever, vomiting, painful swallowing, erythema/exudates on pharynx and tonsils as well as anterior cervical adenitis and the results of the throat swab microscope culture and sensitivity) were presented in frequencies and percentages. A relationship/association among the variables was ascertained using Chi-square statistic. All test statistics were performed at 5% level of significant, hence p < 0.05 was considered statistically significant.

**Ethical consideration:** ethical approval for the study was obtained from the Research and Ethics Committee of the Alex Ekwueme Federal University Teaching Hospital Abakaliki (AE-FETHA). Informed consent was obtained from parents/caregivers of the participants. Assent was obtained from children aged 7 years and above.

## Results

**Socio-demographic analysis:** a total of 3520 patients were seen during the study period. Ten (10) participants that met the study inclusion criteria had antibiotics within 72 hours before presenting to the hospital and were excluded from the study. A total of 150 subjects participated in the study. Table 1 showed the socio-demographic distribution (frequency and percentage) of the studied participants. Variables considered were age, gender, class in school and socio-economic class. Of the 150 participants recruited for the study, 88 were males given a male-to-female ratio of 1.4: 1. The median age of participants was 5.0 years with an inter-quartile range of 3-8 years. Most of the participants were in primary school and majority belonged to upper socio-economic class.

**Table 1:** socio-demographic characteristics of the study participants

Variables	Frequency n=150 (%)
**Age group (years)**	
1-5	80 (53.3)
6-10	57 (38.0)
11-15	12 (8.0)
>15	1 (0.7)
**Gender**	
Male	88 (58.7)
Female	62 (41.3)
**Socio economic class**	
Upper	70 (46.7)
Middle	55 (36.7)
Lower	25 (16.6)

**Univariate analysis:** the relationship between the presence of isolated organisms and symptoms and signs among study group was determined using chi square model as shown in Table 2. The result showed that there was no significant association between the clinical signs and symptoms with the occurrence of bacterial pharyngotonsillitis (p> 0.05).

**Table 2:** relationship between the clinical symptoms and signs of the participants and the laboratory diagnosis of bacterial pharyngotonsillitis

		Organism			
Symptoms/signs		Present (%)	No growth (%)	Chi -square	P-value
Fever	Yes No	45(33.6) 9 (56.3)	89 (66.4) 7 (43.7)	3.183	0.068
Sore-throat	Yes No	34 (32.7) 20 (43.5)	70 (67.3) 26 (56.5)	1.610	0.139
Cough	Yes No	22(32.4) 32 (39.0)	46 (67.6) 50 (61.0)	0.718	0.250
Headache	Yes No	7 (30.4) 47(37.0)	16 (69.6) 80 (63.0)	0.365	0.362
Painful swallowing	Yes No	34 (34.3) 20 (39.2)	65 (65.7) 31 (60.8)	0.347	0.340
Vomiting	Yes No	19 (41.3) 35 (33.7)	27(58.7) 69 (66.3)	0.810	0.231
Abdominal pain	Yes No	4 (44.4) 50 (35.5)	5 (55.6) 91 (64.5)	*0.296	0.415
Tender cervical node	Yes No	30(36.6) 24 (35.3)	52 (63.4) 44 (64.7)	0.027	0.870
Tonsillar/pharyngeal Swelling	Yes No	54 (36.2) 0 (0.0)	95 (63.8) 1 (100.0)	*0.566	0.452
Tonsillar/pharyngeal Exudates	Yes No	32 (36.0) 22 (36.0)	57 (64.0) 39 (64.0)	0.001	0.989
Tonsillar/pharyngeal hyperaemia	Yes No	51 (35.9) 3 (37.5)	91 (64.1) 5 (62.5)	*0.008	0.928
*=Fishers Exact					

**The sensitivity, specificity, positive predictive value and negative predictive value test:** the sensitivity, specificity, positive predictive value and negative predictive value of using clinical features to predict pharyngotonsillitis of bacterial origin were shown in Table 3. Fever, tonsillar swelling and tonsillar/pharyngeal hyperaemia had high sensitivity in predicting pharyngotonsillitis of bacterial origin but their positive predictive values were low.

**Table 3:** the sensitivity, specificity, positive predictive value and negative predictive value of clinical features in predicting bacterial pharyngotonsillitis among participants with identified bacterial organism

Symptoms and signs	Sensitivity (%)	Specificity (%) Predictive Value (%)	Positive	Negative Predictive Value (%)
Fever	(83.3)	(7.3)	(33.6)	(43.7)
Sore throat	(62.9)	(27.1)	(32.7)	(56.5)
Cough	(40.7)	(52.1)	(32.4)	(61.0)
Headache	(12.9)	(83.3)	(30.4)	(63.0)
Painful swallowing	(62.9)	(32.3)	(34.3)	(60.8)
Vomiting	(35.2)	(71.9)	(41.3)	(66.3)
Abdominal pain	(7.4)	(94.8)	(44.4)	(64.5)
Tender cervical node	(55.5)	(45.8)	(36.6)	(64.7)
Tonsillar swelling	(100.0)	(1.0)	(36.2)	(100.0)
Tonsillar/pharyngeal exudate	(59.2)	(40.6)	(36.0)	(64.0)
Tonsillar/pharyngeal hyperaemia	(94.4)	(5.2)	(35.9)	(62.5)

**Figure 1:**
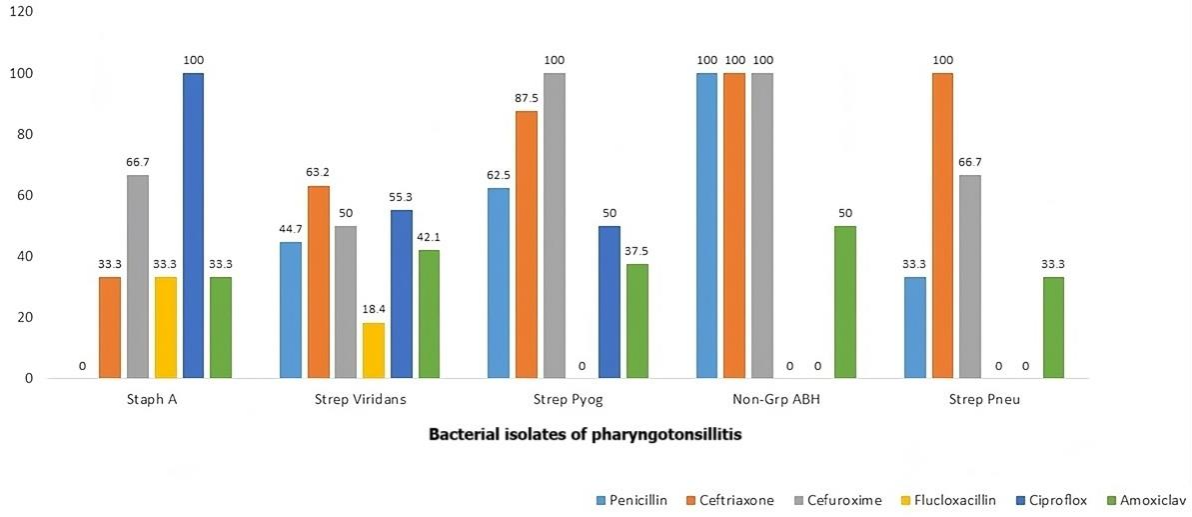
antibiotics susceptibility patterns of the bacterial isolates of pharyngotonsillitis among the participants

**Antibiotics susceptibility:** of the 54 isolated organisms; 38 (70.4%) were *Streptococcal viridans*, 8 (14.8%) *Streptococcal pyogenes, Streptococcal pneumoniae and Staphylococcal aureus* were 3 (5.5%) each and 2 (3.7%) of *non-group A?? haemolytic Streptococcus*, higher susceptibility to cephalosporins (ceftriaxone and cefuroxime) was observed across board when compared to the penicillins and other antibiotics used in the study as shown in Figure 1.

## Discussion

The study observed that the three most frequent symptoms were fever, sore throat and painful swallowing while the more frequent signs were tonsillar enlargement and tonsillar/pharyngeal hyperaemia. Clinical features could not predict bacterial pharyngotonsillitis in this study, even when some symptoms and signs such as fever, tonsillar swelling and tonsillar/pharyngeal hyperaemia had high sensitivity rates; their positive predictive values were low. Furthermore, this study observed that symptoms and signs seen in patients with positive throat culture were also present in those with negative throat culture results. This result was consistent with other studies [4,23-26] that showed the unreliability of using symptoms and signs to predict pharyngotonsillitis of bacterial aetiology. In contrast to Uzodimma *et al*. [27] in Abeokuta, Nigeria who reported cough and exudative tonsils/pharynx to be closely related to bacterial aetiology.

However, in this index study there was an overlap between the clinical presentations of those patients with positive bacteria culture results and those that had negative culture results, suggesting that clinical features alone were unreliable in predicting pharyngotonsillitis of bacterial aetiology. *Streptococcal viridans* was isolated the most in this study followed by *Streptococcal pyogenes*. These two organisms, were observed to be highly sensitive to ceftriaxone and cefuroxime when compared to the commonly used amoxicillin clavulanic acid combination for pharyngotonsillitis. Similar pattern was also seen for *Streptococcal pneumonia* and non-group A *β-haemolytic Streptococcus*. This suggests the effectiveness of cephalosporins in treating pharyngotonsillitis as it displayed sensitivity for a wide range of organisms. Cephalosporins such as ceftriaxone and cefuroxime, are relatively more expensive when compared to the penicillin (penicillin V, amoxicillin clavulanate and flucoxacillin) and as such they may not be the first target for self-medication for an ailment.

Ciprofloxacin (a quinolone) was observed to be highly sensitive to staphylococcal aureus (which was 5.5% of isolated organisms) but resistant to group A *β-haemolytic Streptococcus* and *Streptococcal pneumonia*, hence it may not be ideal for management of pharyngotonsillitis. This observed difference between the sensitivity of the organisms to cephalosporins and penicillin may not be unrelated to possible abuse of the cheaper drugs (penicillin) with the resultant resistance. The pattern of antibiotic susceptibility findings in this study is similar to that documented by previous studies by Sadoh *et al*. [4] in Benin-Nigeria and Janet and Michael [28] in Rochester-New York. In contrast Uzodimma *et al*. [27] in Abeokuta, Nigeria found higher resistance to cephalosporin and erythromycin. The differences noted may not be unrelated to the different strain of streptococcal organisms and their it´s virulence as well as the different types of antibiotics commonly abused in the environment where the studies were conducted.

## Conclusion

In conclusion therefore, an overlap observed between the clinical presentations of the participants with positive throat culture and those without in this study makes clinical features unreliable in predicting pharyngotonsillitis of bacterial origin. Hence routine throat swab for bacteriology culture should be done for all children presenting with features suggestive of pharyngotonsillitis before commencement of antibiotics. However, where such services are not readily available, the use of cephalosporins (cefuroxime and ceftriaxone) are recommended. This study however had limitations. Bacteriologic culture system which utilizes an antibiotic binding resin bead technology to remove effects of antibiotics on culture media [29,30], to enable growth of micro-organism in those who may have taken antibiotics unknowingly from patent medicine vendors before samples were collected from them for culture was not employed in this study due to its non-availability in our facility.

### What is known about this topic

- Some symptoms and signs are predictive of bacterial pharyngotonsillitis;
- Penicillin (penicillin V and amoxicillin clavulanate) are the best antibiotics for the treatment of bacterial pharyngotonsillitis.

### What this study adds

- No clinical symptoms and signs are predictive of bacterial pharyngotosillitis, therefore, throat swab for microscope culture and sensitivity should be done for all suspected cases of pharyngotonsillitis before antibiotic treatment;
- Cephalosporins (cefuroxime and ceftriaxone) are currently the best antibiotic for the treatment of bacterial pharyngotonsillitis.
